# Chronic *Toxoplasma gondii* Infection Modulates Hearing Ability across the Adult Life Span

**DOI:** 10.3390/life14020194

**Published:** 2024-01-29

**Authors:** Stephan Getzmann, Klaus Golka, Peter Bröde, Jörg Reinders, Thura Kadhum, Jan G. Hengstler, Edmund Wascher, Patrick D. Gajewski

**Affiliations:** 1 Leibniz Research Centre for Working Environment and Human Factors at TU Dortmund (IfADo), D-44139 Dortmund, Germany; golka@ifado.de (K.G.); broede@ifado.de (P.B.); reinders@ifado.de (J.R.); hengstler@ifado.de (J.G.H.); wascher@ifado.de (E.W.); gajewski@ifado.de (P.D.G.); 2 Clinic for Psychosomatic Rehabilitation, Mittelrhein-Klinik, D-56154 Boppard-Bad Salzig, Germany; thurajafar@yahoo.com; 3 German Center for Mental Health (DZPG), Partner Site Bochum/Marburg, 44787 Bochum, Germany

**Keywords:** aging, hearing, audiometry, *Toxoplasma gondii*

## Abstract

While several studies have shown associations between hearing disorders and congenital toxoplasmosis, the present study investigated the impact of chronic, latent *Toxoplasma gondii* (*T. gondii*) infection on hearing loss. We used a regression analysis to explore whether latent *T. gondii* infection modulates changes in hearing thresholds over an age range from 20 to 70 years. We analyzed audiometric data of 162 *T. gondii* IgG-positive and 430 *T. gondii*-negative participants, collected in the Dortmund Vital Study (DVS, ClinicalTrials.gov Identifier: NCT05155397), a prospective study on healthy cognitive aging. The regression analysis indicated that latent toxoplasmosis was associated with an accelerated development in hearing loss over the observed age range. Hearing loss was less frequent in IgG-positive than in IgG-negative participants up to the age of about 40 for a low (0.125–1 kHz)-frequency range. For high (2–8 kHz) frequencies, this pattern reversed for ages above 65 years. We discuss these findings on hearing function in the context of a recently proposed model, suggesting that latent toxoplasmosis can differentially affect brain functions across a lifespan.

## 1. Introduction

Latent toxoplasmosis due to infection with the *Toxoplasma gondii* parasite is one of the most common parasitic infections worldwide with a prevalence in about one-third of the adult human population. The prevalence of infection varies between 0 and 100% depending on geographic location ranging from 30% in the USA and United Kingdom, up to 50% in Central and Southern Europe, and up to 80% in Africa and Latin America [1]. Members of the *Felidae* family, including domestic cats, are the definitive hosts of *Toxoplasma gondii*, contaminating the environment with oocysts in their feces. These oocysts are ingested by rodents, as well as other warm-blooded animals like pigs, sheep, and goats. Human infection occurs due to ingestion of oocysts in contaminated vegetables, fruits or water, or upon the ingestion of tissue cysts in raw or undercooked meat [2]. Intracellular tissue cysts are observed in the muscles, heart, and brain, with the majority residing in the brain. This stage of the disease is likely lifelong and cannot be cured [2,3,4,5].

*T. gondii* infection can cause severe disease in immuno-deficient persons and in congenitally infected fetuses if the mother became infected for the first time during pregnancy [6]. However, most *T. gondii* infections are non-specific or even asymptomatic in immuno-competent individuals; albeit, the behavioral changes observed in rodents [7,8,9] led to the question of whether latent infection may compromise the behavior and the neurological or psychiatric status of humans. There are several reports documenting, for example, personality changes, self-directed violence, risk of suicidal tendencies, and several psychiatric disorders such as schizophrenia, obsessive–compulsive disorder, depression, and dysphoria epilepsy in *T. gondii*-infected adults [10,11,12,13,14,15,16,17]. Furthermore, some recent studies found deficits in cognitive functions in *T. gondii* seropositive humans [18,19], particularly in older adults [5,20,21,22,23,24,25]. However, some studies showed a reverse pattern, indicating superior cognitive functions in younger seropositive adults [26,27,28], suggesting differences in neurotransmitter imbalance in infected adults at different ages [26].

While the recent research relates to the consequences of latent toxoplasmosis infection for neurological, mental, and psychological health, effects on sensory functions have so far been rarely described. There is some literature on the relationship between congenital *T. gondii* infection and hearing, suggesting a moderate association between latent toxoplasmosis and sensorineural hearing loss [29,30], but less is known about the potential impact of latent toxoplasmosis on hearing in adults and across the adult’s lifespan. A possible link is suggested by a study on truck drivers in Mexico, in which neurological functions were compared in IgG-positive and IgG-negative individuals and in which the seroprevalence of *T. gondii* infection was higher in drivers with hearing impairment than in those without impairment [31]. Similar results were found in an earlier study [32]. It should be noted that *T. gondii* strains in Latin America have been reported to be more aggressive than the strains in Europe [33]. Furthermore, the results of a European cross-sectional cohort study also showed associations between latent toxoplasmosis and sensory-organ problems. In particular, hearing problems occurred disproportionately more often in *Toxoplasma*-infected than *Toxoplasma*-non-infected men [34].

However, systematic studies of the influence of a latent toxoplasmosis on hearing at different ages are still pending. This seems particularly important since aging is typically associated with a general deterioration in hearing, which is referred to as age-related hearing loss (ARHL, also called Presbycusis; for reviews, see [35,36]). Decline in hearing can be observed, for example, in pure-tone audiometry as an increase in hearing thresholds, which begins in middle age and is particularly evident with high-frequency tones [37]. ARHL mainly affects the inner ear and the cochlear nerve and has many causes [38,39,40], in which infections can be an additional risk factor [41].

The aim of the present study was to examine the influence of latent toxoplasmosis on the development of hearing disturbance for low- and high-frequency tones over the adult life span. For this purpose, cross-sectional data comprising the baseline audiometric examinations from an ongoing, prospective study on healthy cognitive aging with participants aged 20 to 70 years were examined, in which the status of *T. gondii* IgG antibodies were also determined. Based on the proposed model by Colzato et al. [26], improved hearing was expected in *T. gondii* IgG-positive versus -negative young adults and greater deterioration in hearing was expected in *T. gondii* IgG-positive versus -negative individuals at older ages.

## 2. Materials and Methods

### 2.1. Participants

The hearing assessment was part of the Dortmund Vital Study (DVS, Clinicaltrials.gov: NCT05155397), a prospective cohort study on the development of cognitive functions over an age range from 20 to 70 years (for details see [42]). The present sample comprised the baseline examinations with complete data concerning both audiometry and the *T. gondii* IgG antibody status of 592 participants (age range 20–70 years; 366 females, 61.8%). The participants reported to be healthy and free of medication during the testing. All participants gave their written informed consent before any examination was commenced. The study conformed to the Code of Ethics of the World Medical Association (Declaration of Helsinki) and was approved by the local Ethical Committee of the Leibniz Research Centre for Working Environment and Human Factors at TU Dortmund, Dortmund, Germany [42].

### 2.2. Analysis of T. gondii Status

Identification of *T. gondii*-negative and -positive participants without any clinical symptoms of acute toxoplasmosis was performed according to the following procedure: venous blood of all individuals was drawn and tested for *T. gondii*-specific IgG antibodies. The analyses were performed using the *T. gondii* IgG ELISA (IBL International, Hamburg, Germany) according to the manufacturer’s instructions. The ELISA was washed on a hydroFLEX washer and measured on a GENios plate reader system (both TECAN Group Ltd.; Maennedorf, Switzerland). The sensitivity threshold of the *T. gondii* IgG ELISA is 1.04 UI/mL (Siemens Healthcare Diagnostics, 2010). For classification into *T. gondii*-negative and -positive subgroups, participants with low IgG-antibody levels (*N* = 430; serum level < 30 UI/mL) were defined as negative, while those participants with high concentrations of IgG antibodies (*N* = 161; serum level > 35 UI/mL) were defined as positive, according to the manufacturer’s instructions (https://ibl-international.com/en_de/toxoplasma-gondii-igg, accessed on 16 January 2024). Only one participant, who had a concentration of 33 UI/mL was in a gray zone according to the manufacturer’s instructions. This participant was assigned to the positive group.

### 2.3. Audiometry

All participants passed a standard audiometry testing for ten pure-tone frequencies (0.125, 0.250, 0.500, 0.750, 1, 2, 3, 4, 6, 8 kHz) for the left and right ears (Oscilla USB100, Inmedico, Lystrup, Denmark). According to the test protocol, the measurements started at 1 kHz on the left ear. The tone duration was 1 s; the interstimulus interval was 3 s. The maximum response time after the end of the tone was 1 s. The starting level was 0 dB in each frequency, and the level was increased in steps of 5 dB up to a maximum value of 90 dB if there was no response. After a response, the remaining frequencies and both ears were tested in a randomized order.

### 2.4. Data Analysis

For each participant, the individual hearing thresholds were averaged over lower (0.125, 0.250, 0.500, 0.750, 1 kHz) and higher (2, 3, 4, 6, 8 kHz) tone frequencies and over both ears. The mean hearing thresholds were then plotted against the age of the participants. Linear regression fitting in SPSS Statistics (SPSS Version 29; IBM Statistics, Chicago, IL, USA) was then employed to model the change in hearing thresholds as a function of age, independent of the toxoplasmosis status of the participants. Regression analyses were then performed to determine the effect of latent toxoplasmosis on hearing and its influence on the development of hearing with age. By using the PROCESS v 4.2 macro for SPSS (regression model number 1 [43]), we tested whether the effect of age (X; as a continuous variable) on hearing loss (Y) was moderated by *T. gondii* seropositivity (W; as a discrete variable), separately for low and high frequencies. Age was mean-centered prior to analysis, and moderator value(s) defining Johnson-Neyman significance region(s) [44] were computed. Finally, to test whether there was a relationship between hearing loss and latent toxoplasmosis within the seropositive participants, partial correlations between the individual hearing values and the *T. gondii* IgG-antibody concentration of the 162 seropositive participants were conducted, with age being included as a control variable.

## 3. Results

**Figure 1:**
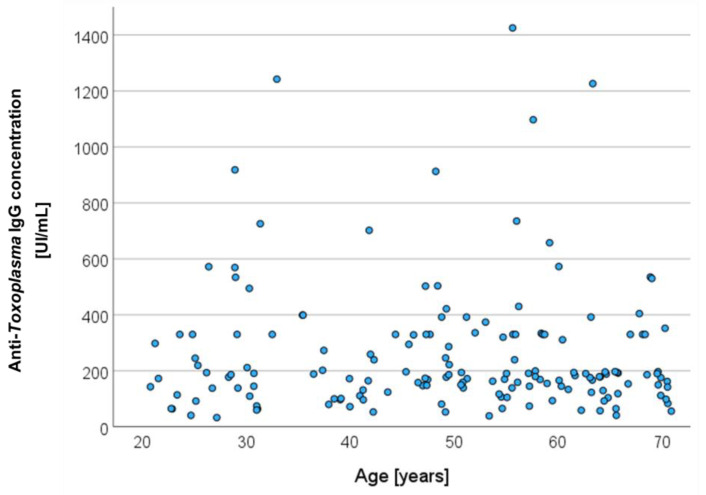
Individual anti-*Toxoplasma* IgG concentration of 162 serum-positive participants as function of age.

Figure 1 shows the individual *T. gondii* IgG-antibody concentration as a function of the participants’ age. Among the 162 serum-positive participants, 45 were younger (27.8%) and 117 were older (72.2%) than 40 years, and 55 had anti-*Toxoplasma* IgG concentrations between 150 and 250 UI/mL (34.0%), and only 18 had concentrations of more than 500 UI/mL (11.1%). There was no significant correlation between anti-*Toxoplasma* IgG concentrations and the age of the participants (*r* = −0.028, *p* = 0.73). The negative and positive subgroups did not differ significantly in gender distribution, according to a chi-squared test, *Χ*^2^ = 1.36, *p* = 0.243 (negative: 158 male, 272 female; positive: 68 male, 94 female). The IgG-positive subgroup was older than the IgG-negative subgroup, according to a *t*-test for independent variables, *t*(592) = 5.270, *p* < 0.001 (negative: mean age 42.8 years, SD 13.8; positive: mean age 49.5, SD 14.5).

**Figure 2:**
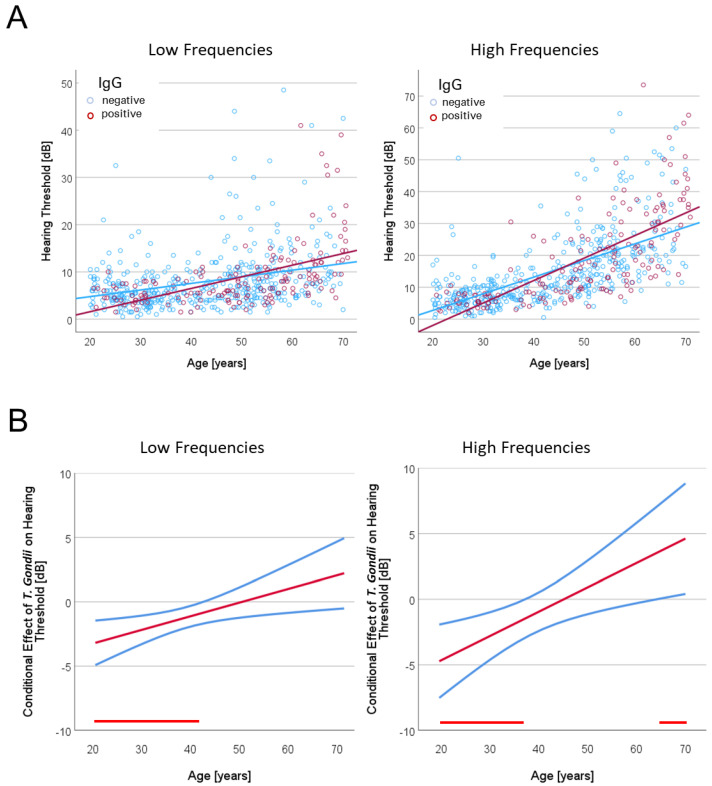
(**A**) Hearing thresholds as function of age for 162 *T. gondii* IgG-seropositive and 430 *T. gondii* IgG-seronegative participants with regression lines, shown separately for the lower (0.125–1 kHz) and higher (2–8 kHz) frequency ranges. (**B**) Johnson-Neyman plot displaying the conditional effects of latent toxoplasmosis on hearing loss as a function of age (red lines) for the lower (0.125–1 kHz) and higher (2–8 kHz) frequency ranges. Upper and lower limits (blue lines) refer to 95% confidence interval (CI) bounds. Age ranges at which latent toxoplasmosis had a significant effect on hearing loss are marked using horizontal red lines. For better comparison with the measured hearing data shown in (**A**), non-centered age values are displayed here.

Plotting individual hearing thresholds as a function of the participants’ age revealed a continuous increase in the hearing threshold with increasing age in both the low- and high-frequency ranges (Figure 2A). In addition, differences in the slopes of the regression lines suggested that this increase was more pronounced in the IgG-positive subgroup than in the IgG-negative subgroup.

For the low-frequency range, the moderation analysis indicated that the overall model was significant, *F*(3, 588) = 30.024, *p* < 0.001, predicting 15.23% of the variance. The analysis did not indicate an effect of latent toxoplasmosis on hearing loss, *t* = 1.249, *p* = 0.212, 95% CI [−1.510, 0.336], but a highly significant effect of age, *t* = 6.021, *p* < 0.001, 95% CI [0.094, 0.186]. In addition, there was a significant interaction of age and latent toxoplasmosis on hearing loss, *F*(1, 588) = 6.557, *p* = 0.011, 95% CI [0.025, 0.188], ΔR^2^ = 1.10%. Thus, the decline in hearing was stronger in the IgG-positive (0.246 dB per year) than in the IgG-negative group (0.140 dB per year). The Johnson-Neyman interval showed that the effect of latent toxoplasmosis on hearing loss was significant in the age range below 42.2 years, in which *T. gondii*-positive participants had lower estimated thresholds suggesting better hearing performance than *T. gondii*-negative participants, whereas the effect of latent toxoplasmosis was not significant at older age (Figure 2B).

For the high-frequency range, the moderation analysis also indicated an overall significant model, *F*(3, 588) = 132.25, *p* < 0.001, predicting 45.35% of the variance. Results further showed a significant effect of age on hearing loss, *t* = 14.623, *p* < 0.001, 95% CI [0.452, 0.593], but no main effect of latent toxoplasmosis, *t* = 0.276, *p* = 0.783, 95% CI [−1.822, 1.373]. However, latent toxoplasmosis moderated the effect between age and hearing loss significantly, *F*(1, 588) = 8.368, *p* < 0.004, 95% CI [0.059, 0.308], ΔR^2^ = 0.89%, indicating a stronger decline in hearing in the IgG-positive (0.706 dB per year) than IgG-negative group (0.523 dB per year). The Johnson-Neyman interval indicated two age ranges in which the effect of latent toxoplasmosis on hearing loss was significant (Figure 2B): in the age range up to 37.9 years, the estimated hearing loss was lower in *T. gondii*-seropositive participants than in *T. gondii*-negative participants, whereas this pattern reversed for age above 65 years.

The calculation of the partial correlations between hearing loss and the *T. gondii* IgG concentration of the 162 seropositive participants (with age as control variable) showed no associations, neither for the low-frequency range, *r* = 0.008, nor for the high-frequency range, *r* = 0.006, both *p* > 0.90.

## 4. Discussion

The results of the audiometry tests showed a typical decline in hearing ability with age, which is referred to in the literature as age-related hearing loss (ARHL, also known as Presbycusis) and which is one of the most widespread sensory impairments in old age (for reviews, see [35,36]). Studies have shown changes in hearing ability from around the third decade of life, with only predominantly high-frequency hearing being initially affected. With increasing age, impairments occur at wider frequency ranges, which also impact the speech intelligibility and can have serious consequences for everyday life and the well-being of older people [45]. Accordingly, a milder progression of hearing loss was found in the lower frequency range than in the higher range. While the overall trend showed a linear decrease with age, there were individuals with a more pronounced decrease in hearing performance and—as often observed with increasing age—an increasing variance between individuals (cf. Figure 2A). Overall, this progression of reduced hearing ability with age corresponds to that of previous studies. For example, the cross-sectional analysis of the Baltimore Longitudinal Study of Aging showed an almost linear decrease in hearing in the age range from 25 to 90 years in the range from 0.5 to 4 kHz [46,47], while the change in other studies corresponded more to a non-linear course in which the deterioration in hearing accelerated with increasing age [48,49]. ARHL is mainly associated with the functional loss of sensory and neural functions [38,39,40]. The mechanisms of this are–although not yet fully understood–quite diverse, with various risk factors playing a role [41]. In addition to age, these include gender, ethnicity, environment (e.g., noise exposure), lifestyle (e.g., smoking, nutrition), comorbidities (e.g., hypertension, diabetes), and genetic predisposition. While some of these risk factors are associated with age, many effects (like exposure to intense noise) can occur regardless of a person’s age. This is also true for infections that might cause hearing loss (for review, see [50]). One extreme example is the incidence of sudden sensorineural hearing loss (SSNHL), which has been related to viral infection within the cochlea as well as immune-mediated mechanisms [51].

The present study showed a modifying influence of latent toxoplasmosis on the development of hearing loss with age. The increase in hearing threshold was steeper in the IgG-positive group than in the IgG-negative group in both the low- and high-frequency ranges. The overall effect was—in view of the explained variance—very small and will, therefore, probably become of practical relevance for the hearing of the individual only when considered in combination with other risk factors mentioned above. However, it could contribute to understanding the mechanisms of latent toxoplasmosis. A latent *T. gondii* infection has so far been associated with numerous phenomena, such as mental and psychological functions, while the effects of latent toxoplasmosis on hearing have not yet been systematically investigated to the best of our knowledge. Only a few previous studies have reported an association between latent toxoplasmosis infection and hearing [31,32,34]. In a study with truck drivers in Mexico, the seroprevalence of latent *T. gondii* infection was higher in persons with hearing impairment than in those without this impairment [31]. It should be noted that the participants in this study were between 30 and 50 years old, with only a few younger than 30. Furthermore, an exploratory cross-sectional study on the health status and the incidence of diseases in 333 IgG-positive and 1153 IgG-negative individuals with an average age of about 35 years revealed associations between latent toxoplasmosis and a number of health-related variables including problems with sensory organs in men [34]. In particular, differences in the incidence of hearing loss between IgG-positive and -negative groups were found.

In our study, however, the accelerated increase in the hearing threshold over the age range did not only result in more impaired hearing in older IgG-positive than IgG-negative individuals (which was also only found in the high-frequency range which is more critical for this group), but also in younger IgG-positive individuals with a less steep increase in hearing threshold than IgG-negative individuals, in both frequency ranges. A very similar effect was observed in a study on odor perception with young participants (average age 28 years), in which IgG-positive men performed better than their IgG-negative counterparts [52]. While this pattern seems unexpected, it nicely corresponds to the model proposed by Colzato et al. [26] on the influence of latent toxoplasmosis on cognitive functions. This model was conceptualized after observing different outcomes on cognitive tasks in young and older adults: while IgG-positive older adults show a consistent decline of cognitive functions [20,21,23], other studies showed less-consistent effects or even better performance in IgG-positive young adults ([27,53]; for review, see [18]). This model proposes a shift in the balance between increased dopamine synthesis induced by *T. gondii* infection and neurodegeneration and inflammation leading to reduction of dopamine synthesis in older age, which is crucial for sufficient cognitive functioning.

No dose–response relationship was observed, that is, no correlation was found between the degree of the infection (the concentration of antibodies at the time of the examination) and the hearing loss. However, it is critical to note that the duration of chronic infection could not be determined. It could, therefore, be, for example, that a 70-year-old has only recently had an infection or has had it since early childhood and the effect of this has been affecting hearing for just as long. The observed correlation between age and prevalence of latent toxoplasmosis infection should also be noted: On the one hand, it seems plausible that the probability of infection accumulates over time, leading to a higher percentage of IgG-positive persons in an older population, which has also been shown in previous studies [54]. On the other hand, there is evidence from epidemiological studies suggesting that the seroprevalence only increases up to about 40 years in men and 50 years in women. At an older age, the rate of positive to negative seroconversions could predominate over the infection rate [55,56]. While remaining infected, due to immunosenescence, the levels of IgG antibodies in some individuals could fall below the threshold of the given test. Thus, assuming some of the *Toxoplasma*-negative rated older individuals to be actually *Toxoplasma* positive, the moderating effect of toxoplasmosis on hearing loss could be even stronger than observed here.

One limitation of the present study is, therefore, that such cohort effects cannot be considered due to the cross-sectional design. Yet, the Dortmund Vital Study [42] is conceptualized as a longitudinal study with follow-up measures, which make it possible to analyze such cohort effects in the future. Furthermore, it should be noted that, although the findings showed the modulating effect of latent toxoplasmosis, the question of the possible underlying mechanism is still open and requires further investigation. Future studies might consider additional parasite factors affecting toxoplasmosis severity, e.g., by genotyping *T. gondii* strains. Moreover, an intriguing question is related to the possible generalization of this pattern to other domains such as cognition.

## 5. Conclusions

The present study evaluated hearing ability across the adult life span and its modulation by latent toxoplasmosis. The results showed that the annual rate of hearing loss is different for low and high frequencies, and that both frequency ranges are differently affected in seropositive and seronegative individuals. The most important observation was a reversal of hearing ability in middle age as a function of infection, suggesting more efficient auditory processing in seropositive young adults and a greater decline in older age than in seronegative adults. The findings suggest that the effects of latent toxoplasmosis should be considered separately in different age groups.

## Data Availability

The data that support the findings of this study are available from the corresponding author upon reasonable request.
